# Long-Term Fluctuations in Circalunar Beach Aggregations of the Box Jellyfish *Alatina moseri* in Hawaii, with Links to Environmental Variability

**DOI:** 10.1371/journal.pone.0077039

**Published:** 2013-10-23

**Authors:** Luciano M. Chiaverano, Brenden S. Holland, Gerald L. Crow, Landy Blair, Angel A. Yanagihara

**Affiliations:** 1 Kewalo Marine Laboratory, Pacific Biosciences Research Center, University of Hawaii at Mānoa, Honolulu, Hawaii, United States of America; 2 Center for Conservation Research & Training, Pacific Biosciences Research Center, University of Hawaii at Mānoa, Honolulu, Hawaii, United States of America; 3 Waikiki Aquarium, University of Hawaii at Mānoa, Honolulu, Hawaii, United States of America; 4 Ocean Safety and Lifeguard Services, City and County of Honolulu, Honolulu, Hawaii, United States of America; 5 Bekesy Laboratory, Pacific Biosciences Research Center, and Department of Tropical Medicine, John A. Burns School of Medicine, University of Hawaii at Mānoa, Honolulu, Hawaii, United States of America; University of Wales Swansea, United Kingdom

## Abstract

The box jellyfish *Alatina moseri* forms monthly aggregations at Waikiki Beach 8–12 days after each full moon, posing a recurrent hazard to swimmers due to painful stings. We present an analysis of long-term (14 years: Jan 1998– Dec 2011) changes in box jellyfish abundance at Waikiki Beach. We tested the relationship of beach counts to climate and biogeochemical variables over time in the North Pacific Sub-tropical Gyre (NPSG). Generalized Additive Models (*GAM*), Change-Point Analysis (*CPA*), and General Regression Models (*GRM*) were used to characterize patterns in box jellyfish arrival at Waikiki Beach 8–12 days following 173 consecutive full moons. Variation in box jellyfish abundance lacked seasonality, but exhibited dramatic differences among months and among years, and followed an oscillating pattern with significant periods of increase (1998–2001; 2006–2011) and decrease (2001–2006). Of three climatic and 12 biogeochemical variables examined, box jellyfish showed a strong, positive relationship with primary production, >2 mm zooplankton biomass, and the North Pacific Gyre Oscillation (NPGO) index. It is clear that that the moon cycle plays a key role in synchronizing timing of the arrival of *Alatina moseri* medusae to shore. We propose that bottom-up processes, likely initiated by inter-annual regional climatic fluctuations influence primary production, secondary production, and ultimately regulate food availability, and are therefore important in controlling the inter-annual changes in box jellyfish abundance observed at Waikiki Beach.

## Introduction

Jellyfish (cubozoans, hydrozoans and scyphozoans) are conspicuous, ecologically important constituents of coastal and oceanic systems. In the last three decades, jellyfish have received growing attention due to fluctuation in abundance often resulting in population explosions (e.g., blooms) in marine ecosystems worldwide, and frequently interfering directly with human activities [1]. When abundant, jellyfish cause widespread problems by clogging fishing nets [2], [3], causing fish mortality in aquaculture pens [4], [5], clogging intake screens in power generation and desalination plants [6], and impact tourism by stinging swimmers [7].

Predicting changes in jellyfish aggregations over time has been a difficult task due to a number of factors, including difficulties associated with sampling [8], scarcity of historical records and long-term time series datasets [1], [9], and the unusual characteristics of their life cycle [10], [11]. Many hydrozoans and scyphozoans, and all cubozoans, have complex life cycles including a benthic sessile polyp phase and a planktonic medusa phase [12]. Under favorable conditions, polyps can asexually produce large numbers of new polyps and ephyrae (i.e., immature medusae) [13], which can quickly grow into adult medusae [14]. Under adverse conditions, polyps can form dormant cysts [15] and medusae can also stop somatic and reproductive growth [16], [17] until favorable conditions return [18], [19]. This remarkable plasticity makes jellyfish suited to highly variable environments, and can result in large temporal and spatial fluctuations in abundance at various time scales [20].

Although many jellyfish populations appear to respond to anthropogenic processes, such as coastal eutrophication, overfishing, translocation of species, benthic-trawling and increased substrate availability for polyp settlement [1], [19], [21], [22], [23], [24], there is increasing evidence that jellyfish populations are affected by large-scale climate variation and regional environmental conditions associated with climate fluctuations [25], [26], [27]. In the Irish Sea, 68% of jellyfish abundance was explained by variation in the North Atlantic Oscillation, sea surface temperature, zooplankton biomass, and precipitation [28]. Within the northern California current the highest catches of medusae correlated with cool temperatures during spring-summer and low winter-summer runoff of the Columbia River, and negative anomalies of the Pacific Decadal Oscillation (PDO) [29].

Understanding the relationships among large-scale climate forces and associated regional environmental variables with fluctuations in jellyfish populations over time is crucial to explaining and predicting trends in jellyfish abundance on a global scale [9], [30]. Long-term studies on climate fluctuations and jellyfish have increased in the last decade. However, most of these studies have taken place in highly productive, temperate, coastal environments, and only a few attempts have been made to assess trends in jellyfish abundances in oligotrophic, open-ocean gyres [31], [32]. In addition, long-term studies of jellyfish abundance and climate exist for only a few species of scyphozoans, hydrozoans, and ctenophores [1], and no studies exist for cubozoan species. This is somewhat surprising as box jellyfish are among the most venomous animals in the world [33]. Quite often, their habitat overlaps with areas of human recreation resulting in dangerous encounters due to painful, even lethal stings [34], [35], causing beach closures at various global localities [33].

We have assessed long-term trends in abundance of box jellyfish and investigated the role of environmental conditions in an oligotrophic environment. The North Pacific Sub-tropical Gyre (NPSG) is the earth's largest contiguous oligotrophic biome, extending from 15°N to 35°N and from 135°E to 135°W, encompassing the entire Hawaiian Archipelago [36]. *Alatina moseri* Gershwin, 2005 (previously *Carybdea alata* Reynaud, 1830) is a tropical box jellyfish (Cubozoa) with a complex metagenic life cycle involving a benthic polyp and pelagic medusae [37]. On the Hawaiian Island of Oahu, reproductive cubomedusae have been observed near shore for a 2–4 day period 8–12 days after each full moon (i.e., during the waning crescent phase) along Waikiki beach since 1994 [38], and sporadically along beaches of other leeward Oahu bays. Box jellyfish aggregations often cause mass stinging events and beach closures [39]. Occasional circalunar sightings of box jellyfish on the south shore of Oahu (Waikiki Beach and surrounding areas) were first noted the late 1980s [38], and the timing of this monthly phenomenon has remained consistent. However, questions regarding whether the number of box jellyfish arriving at the beach has changed over time, or if fluctuations are affected by environmental conditions, have not been previously addressed. We have conducted a 14-year daily beach census of *Alatina moseri* medusae occurrence in leeward Oahu focused on Waikiki in order to: 1) assess seasonal patterns in abundance, 2), determine whether abundance of box jellyfish arriving at the beach has changed over time, and 3) explore potential links in variance of box jellyfish abundance at the beach to fluctuations in environmental conditions within the NPSG. This approach will provide useful information regarding the ecology of the Cubozoa, insights into jellyfish responses to environmental conditions in open-ocean, subtropical gyres, and will ultimately enhance our ability to predict the extent of onshore influxes of venomous jellyfish species in areas where they impose a health hazard.

## Methods

### Box jellyfish abundance and bell height at Waikiki Beach

**Figure 1:**
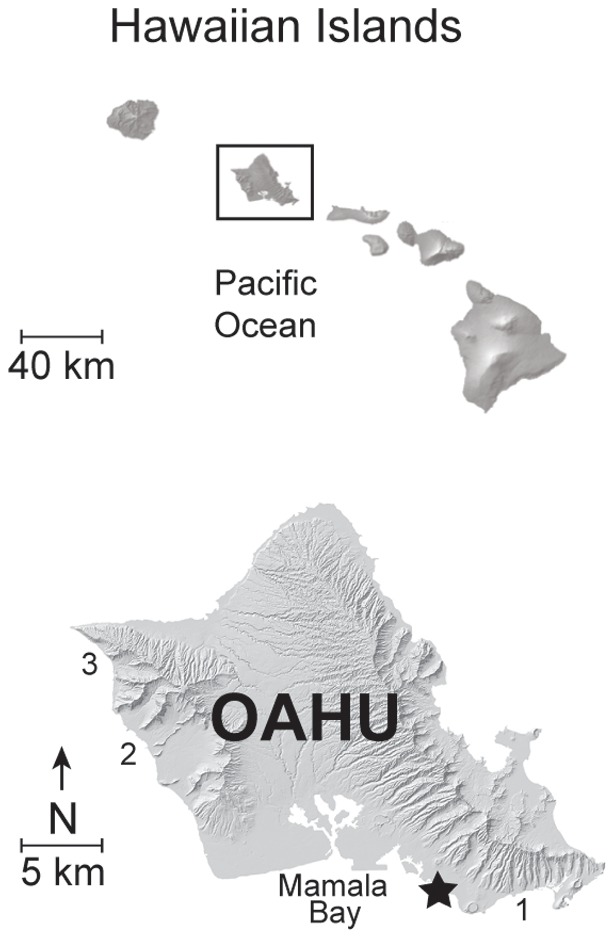
Map of the island of Oahu showing study area along Waikiki Beach. Box jellyfish, *Alatina moseri*, were collected monthly from 1998–2011 at Waikiki Beach, which is represented by the star symbol. Numbers indicate beach areas along leeward Oahu where *A. moseri* medusae arrive sporadically: 1) Hanauma Bay, 2) Pokai Bay, 3) Yokohama Bay.

Box jellyfish beach surveys were carried out monthly at Waikiki Beach, Oahu, Hawaii (21° 16.2′ N, 157° 49.4′ W; Figure 1) by University of Hawaii, Ocean Safety and Lifeguard Services (OSLS) personnel, and volunteers from January 1998 through December 2011. Waikiki Beach, a ∼3 km beach on the leeward end of Mamala Bay along the south shore of Oahu (Figure 1), was chosen due to the consistent majority prevalence of box jellyfish arrivals in this area, and its popularity as a tourist destination; it is also the most visited beach in the state of Hawaii. Waikiki Beach was patrolled and checked for the presence of box jellyfish on a daily basis. When *Alatina moseri* medusae were present on or near the beach (typically 8–12 days after each full moon), surveys were initiated. Box jellyfish arrive at Waikiki Beach exclusively during the early morning ebbing tide. Thus, surveys were typically performed from 0200 to 0700 in order to ensure that observers were present for the duration of the influx. Surveys consisted of walking and wading into the shallow water, along the designated portion of shoreline (see below), in one direction. When one end of the section was reached, the search proceeded in the opposite direction. This protocol was repeated until all jellyfish that arrived during a given influx were collected and counted. Individuals were recently beached or swimming in the shallow waters at the time of collection, and visualized with head-lamps and/or flashlights, then collected by hand. Since arrival of medusae to Waikiki Beach can occur on multiple consecutive nights, searches were carried out daily until no medusae were found. Abundance of box jellyfish at the beach was determined by standardizing: 1) *Area searched*: all box jellyfish included in this study were collected from a 400 m portion of Waikiki, up to 1.5 m deep (Figure 1), via 14 years of monthly surveys. The study area was selected because of regular arrival of box jellyfish each month (hereafter referred to as Waikiki Beach). The geographic distribution of box jellyfish arrival on Oahu is not uniform around the island, such that jellyfish counts from Waikiki Beach cannot be extrapolated to other Hawaiian beaches. During the study period, OSLS personnel monitored box jellyfish influxes in other beaches along leeward Oahu, such as Hanauma Bay, Pokai Bay, and Yokohama Bay (Figure 1), and only Mamala Bay (Waikiki Beach) showed consistent monthly influx events (AAY, unpubl. data). 2) *Number of participants*: All box jellyfish medusae were collected within the designated area by 2–4 people. Bell height was recorded for all individuals collected monthly during 2001. As *Alatina moseri* medusae arriving at the beach are relatively large adult individuals (mean bell height = 64 mm, *SD*: 9; Table S1 & Figure S1 in File S1), they can be easily spotted and collected in the relatively small study area. Adult specimens of *A. moseri* can be easily distinguished from adults of the other two cubozoan species occurring in Hawaii, *Carybdea sivickisi* [40] and *Carybdea arborifera* [41]. Although not all individuals counted during this 14 year study were measured, all were examined and confirmed to be adult *A. moseri*. This research did not require collection permits, since the box jellyfish *A. moseri* is neither an endangered nor a protected species.

### Climate data

Climatic phenomena within the subtropical North Pacific were described using bi-monthly indices of the Multivariate ENSO Index (MEI), and monthly indices of the Pacific Decadal Oscillation (PDO) and the North Pacific Gyre Oscillation (NPGO), for 1998–2011. The MEI is used to evaluate the variability in ENSO forcing, and it is obtained as the first unrotated principal component of six observed variables (sea-level pressure, zonal and meridional components of surface winds, sea surface temperature, surface air temperature, and total cloud cover) confined to the tropical Pacific [42]. This index is available online from NOAA Climate Diagnostic Center at http://www.cdc.noaa.gov/people/klaus.wolter/MEI/table.html. MEI values refer to El Niño and La Niña conditions, respectively. The PDO forcing emerges as the first mode of sea surface temperature (SST) and sea surface height (SSH) anomalies in the North Pacific Ocean poleward of 20° N [43], and is strongly correlated with variability in atmospheric circulation around the Aleutian low pressure system. Positive and negative PDO values refer to warm and cool phases, respectively. The PDO indexes were obtained from the Joint Institute for the Study of the Atmosphere and Ocean website at http://jisao.washington.edu/pdo/PDO.latest. The NPGO index represents the second principal component of sea surface height variability in the Northeast Pacific and, as such, is strongly correlated with fluctuations in the intensity of the geostrophic circulation in this region and its signal is strong south of 38° N [44]. The NPGO indices are available online from Dr. Emanuele Di Lorenzo's website at http://www.o3d.org/npgo/npgo.php. Positive and negative index values refer to more and less intense gyre circulations, respectively.

### Biogeochemical data

Thirteen biogeochemical variables collected at station ALOHA (A Long-term Oligotrophic Habitat Assessment), 22°45′ N, 158°W, were downloaded from the HOT Data Organization and Graphical System (HOT-DOGS©) website at http://hahana.soest.hawaii.edu/hot/hot-dogs/interface.html. The location of station ALOHA was carefully chosen to be representative of biogeochemical processes characteristic of the North Pacific Subtropical Gyre (NPSG) [45], which encompasses all main Hawaiian Islands. Variables included: sea surface temperature (SST, °C), surface salinity (S, ppt), particulate nitrogen (PN mmol m^−2^), and carbon (PC, mmol m^−2^) concentrations, chlorophyll a concentration (CHa, mg m^−2^), *Prochloroccocus* (PL, #×10^11^ m^−2^), eukaryotes (EU, #×10^11^ m^−2^) and heterotrophic bacteria (HB, #×10^11^ m^−2^) abundance, primary production (PRI, mgC m^−2^ day^−1^) and zooplankton biomass (ZO, g DW m^−2^), which included “day” and “night” zooplankton biomass. Each group was separated into two range sizes: <2 mm and >2 mm (for zooplankton collection and processing methods, see [46]). We integrated values (100 m to the surface, except zooplankton biomass, which was integrated from 160 m to the surface [46]) for all variables except SST and S, for which we obtained surface (0–5 m) monthly means. Variables downloaded spanned the study period from January 1998 to December 2010. Information and description of protocols used for sampling, experimentation and analysis are available from the HOT-DOGS© website at http://hahana.soest.hawaii.edu/hot/hot-dogs/interface.html.

### Local weather data

Weather conditions for the Waikiki Beach area were examined using seven local weather parameters, acquired from the National Climatic Data Center (NCDC) at http://www.ncdc.noaa.gov/including: daily air temperature (°C), rainfall (mm), cloud cover (%), humidity (%), atmospheric pressure (mm Hg), wind speed (km/hr), and wind direction (degrees). Daily values for the period from January 1998 to December 2011 were used to calculate monthly and annual means. For monthly and annual wind direction, we used the method described by Gilhousen (1987) [47] to obtain daily “x” and “y” vector components of wind direction. Average x and y values were then calculated per month and per year, and the resultant wind direction (i.e., vector) was derived from arc-tangent (y/x). Air temperature and rainfall data were obtained from the weather station Waikiki 717.2 (USC00519397: 21° 16′ 17.8” N, 157° 48′ 57.6” W), the remaining parameters were recorded at the weather station at Honolulu International Airport (USW00022521: 21° 19′ 26 N, 157° 55′ 46” W).

### Statistical analysis

#### Box jellyfish seasonality, medusa size, and long-term trends of abundance

Prior to analysis, beach counts were log_10_-transformed to achieve normal distribution (*Kolmogorov-Smirnov* test, *d* = 0.74, *p* = 0.25). In order to evaluate seasonality of box jellyfish abundance, monthly data from all years were combined into 12 months, and a one-way ANOVA was used to test for significant differences among months. An additional one-way ANOVA was applied to assess seasonality in the bell height of *Alatina moseri* medusae collected throughout 2001. For trend analysis, we used monthly data and mean annual abundances (log-transformed). Potential long-term trend changes in box jellyfish abundance (i.e., net increase or decrease) were assessed using *General Linear Regression Models* (*GRM*). *Generalized Additive Models* (*GAM*) were used to assess potential temporal trends (monthly and annual scales) in the dataset. The *GAM* is a nonlinear regression technique that uses non-parametric smoothing functions (i.e., cubic splines) to model the relationships among response and forcing variables [48], [49], [50]. An appealing feature of *GAMs* is that they do not assume a functional form for the fitted curve *a priori*. Instead, each predictor variable in the model is separated into sections delimited by “knots”, and polynomial functions are fit to each section separately, revealing underlying trends in the observed data [48]. Once periods of decrease and increase in jellyfish abundance were determined by *GAM*, *GRM* models were used to test whether the linear slope deviates significantly from zero within each period. Trend analysis was carried out using the statistical software package Statistica© v7 with the associated distribution set to “normal” and the default smoothing function (i.e., cubic spline). Non-linear *r* – values were tested for significance at *α* = 0.05.

To further evaluate whether significant shifts in monthly abundances (log-transformed) occurred within the periods determined by the *GAM*, we performed a change-point analysis (*CPA*) using the software Change-Point Analyzer © v2.3 [51]. The *CPA* utilizes a combination of time varying cumulative sum charts (*CUSUM*) and bootstrapping to detect significant shifts in the mean of time-ordered datasets, providing number of shifts, direction of each change, and the time of each shift with respective confident levels and confident intervals. This procedure tests the null hypothesis of “no change” across the dataset. Number of bootstrap samples was set to 2,000, without replacement. The confidence level for identifying candidate changes was set at 90%; however, a regime shift was considered to be significant only when confidence levels were higher than 95%, and confidence intervals around a significant change were set at 99%.

#### Climate indices, biogeochemical variables, and weather parameters versus box jellyfish abundance

Since non-stationary effects are the rule rather than the exception in ecology, box jellyfish abundance, climate, biogeochemical variables, and weather parameters were detrended (except wind direction) by linearly regressing each variable (log_10_-transformed) against time (monthly scale) to remove temporal trends, and the residuals were saved for subsequent analyses [52]. No significant autocorrelations were detected in the dataset (*Chelton Method*; [53]). Mean annual residual values were then obtained. Potential associations among detrended variables (monthly and annual scales) were evaluated using *GAM* with a univariate approach, with jellyfish abundance as the response variable. When a relationship was detected as non-significant by the *GAM* (non-linear *p*>0.05), a *GRM* was used. The *GAM* and *GRM* analyses were performed using Statistica v7. In order to test for delayed response, or lag of jellyfish to environmental variables, we performed a *Cross-correlation* analysis considering ±1-year (annual scale). All cross-correlation analyses were carried out in Minitab v13. In order to test the potential association between box jellyfish counts and wind direction (monthly and annual means), *Circular-Linear* correlation analyses were performed using the circular statistics software Oriana© v.4.

## Results

### Seasonality, medusa size, and long-term trends of box jellyfish abundance at Waikiki Beach

Abundance of *Alatina moseri* medusae at Waikiki Beach did not vary significantly among months, indicating no seasonality (*F_11, 156_* = 0.31, *p* = 0.98). During this study, *A. moseri* medusae arrived at Waikiki Beach 8–12 days after each full moon (i.e., during waning crescent phase of the moon). Arrivals of box jellyfish to the beach happened on 1–4 consecutive nights, with the exception of February 2002 when medusae arrived on 9 consecutive nights. Circalunar aggregations frequently consisted of 100–1000 box jellyfish (65% of the counts), and arrivals of less than 100 individuals, or more than 1,000, occurred 28% and 7% of the time, respectively. A total of 66,605 medusae were counted between January 1998 and December 2011, with an average of 396.5 individuals (range: 5–2,365; median: 271.5) arriving 8–12 days after each full moon and 4,757 per year (range: 2,155–8,696; median: 4,773) (Table S2 in File S1).

The bell height of individuals (*n = 4228*) measured during beach surveys in 2001 varied significantly among months (*F_1, 11_* = 70, *p*<0.0001); however differences in mean bell height throughout the year did not exceed 1.2 cm. The significant differences detected by the ANOVA were due to the consistently small variation in medusae size observed within months (Table S1 & Figure S1 in File S1).

**Figure 2:**
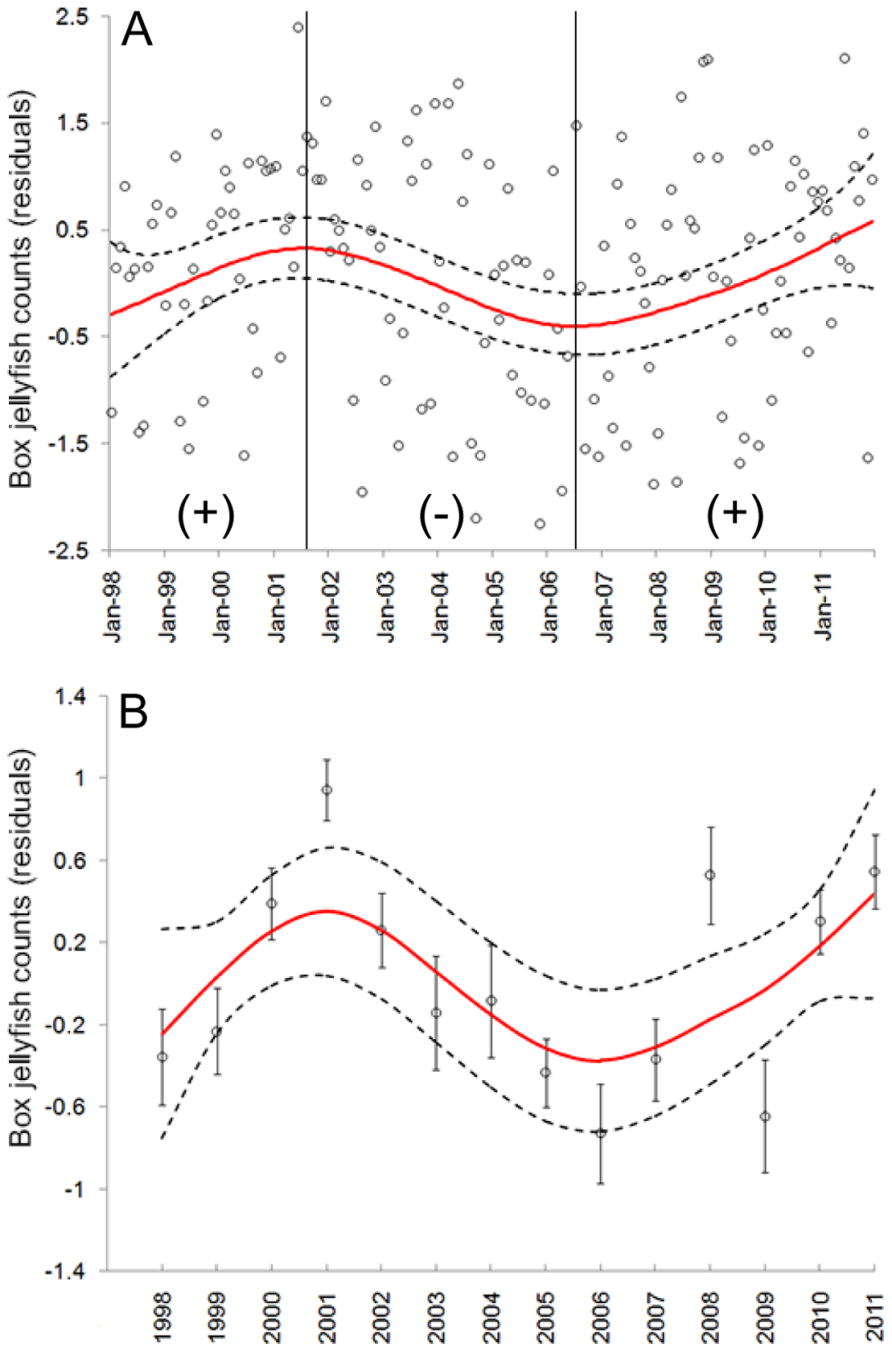
Long-term changes in abundance of box jellyfish at Waikiki Beach. Trends of box jellyfish abundance (solid line) over time was determined by the Generalized Additive Model (*GAM*) using (A) monthly and (B) annual mean counts. Data were log-transformed and detrended before analysis. Dashed lines indicate 95% confidence limits. Vertical solid lines indicate periods of significant (*Linear regression*, *p*<0.05) increase (+) or decrease (−) in box jellyfish counts. Bars show standard errors.

The *GRM* did not reveal a long-term trend in box jellyfish abundance at the beach (i.e., a net increase or decline over time); the linear slope of box jellyfish abundance (log-transformed) did not deviate significantly from zero during the period of study (1998–2011: Slope: 0.0012±0.002; *p* = 0.51; *n* = 168). However, the *GAM* indicated that box jellyfish abundance co-varied with time in a cyclical, non-linear fashion, explaining 12% of the variation in monthly abundance (non-linear *p* = 0.003), and 53% of the variation in mean annual abundance (non-linear *p* = 0.01) during the 14-year study, respectively (Figure 2A & 2B). Thus, based on periods determined by the *GAM*, box jellyfish aggregations: 1) significantly increased from 1998 to 2001 (*GRM*, *r = *0.46, *r^2^* = 0.26; *p*<0.01), 2) significantly decreased from 2001 to 2006 (*GRM*, *r = *−0.45, *r^2^* = −0.21, *p*<0.001), and 3) then significantly increased until the end of data collection in 2011 (*GRM*, *r = *0.29, *r^2^* = 0.18; *p*<0.001). Thus, the 14-year dataset was characterized by four intermittent periods of increase and decrease in box jellyfish abundance, each period lasting approximately four years (Figure 2A).

**Figure 3:**
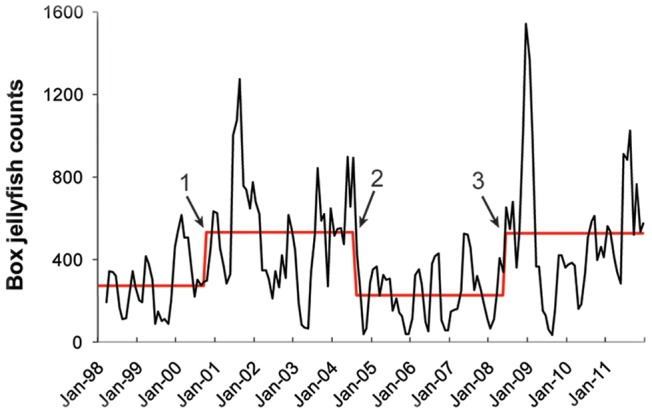
Monthly time-series of box jellyfish counts at Waikiki Beach. A three-month moving average smoothing approach was used to enhance visualization of the trend, depicted by the solid back line. The solid red line indicates mean box jellyfish beach counts during periods of no significant change determined by the Change Point Analysis (CPA). Three significant regime shifts in jellyfish numbers occurred during the 14-year period of study, corresponding to the periods previously identified by the *GAM* analysis: 1) 2000, 2) 2004, and 3) 2008. Dataset had no zeroes.

Change-Point Analysis (*CPA*) detected three significant shifts in average abundance throughout the 14-year period, each one within periods of increase or decrease previously detected by the *GAM*. The first shift was detected in 2000 (October ±6 months, 100% confidence level), with average abundance increasing from 273 to 534. The second shift was detected in 2004 (August ±6 months, 100% confidence level), with average abundance decreasing from 534 to 227, and the third one was detected in 2008 (June ±8 months, 98% confidence level), with increased average beach abundance from 227 to 526 (Figure 3).

### Relationships between box jellyfish counts and climate indices, biogeochemical variables, and weather parameters

**Figure 4:**
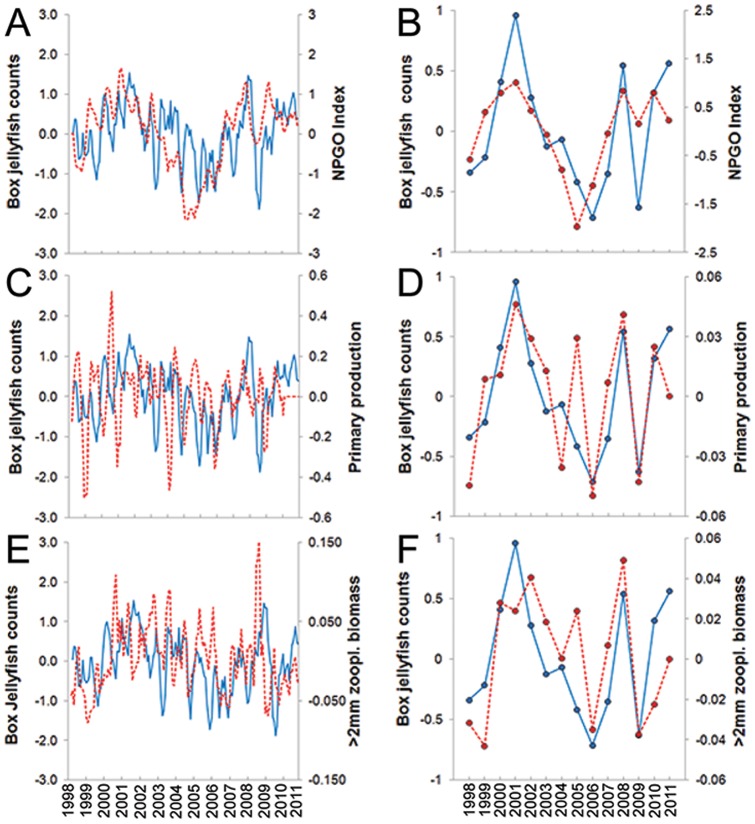
Time-series of climatic and biogeochemical variables with box jellyfish beach counts at Waikiki. Log-transformed, detrended variables are shown, Y-axes are residual values. Graphs A, C, and E represent monthly time-series (three-month average smoothing), while graphs B, D, and F represent annual means. Solid blue lines represent box jellyfish numbers, dashed red lines represent climate and biogeochemical variables, including NPGO (A, B), primary production (C, D), and zooplankton biomass (E, F). Note different scale in the Y-axis of variables between monthly and annual analyses. Error bars were not included in order to enhance visualization. Biogeochemical variables were obtained at Station ALOHA, from the Hawaiian Oceanographic Time-series program (HOT) at http://hahana.soest.hawaii.edu/hot/.

**Figure 5:**
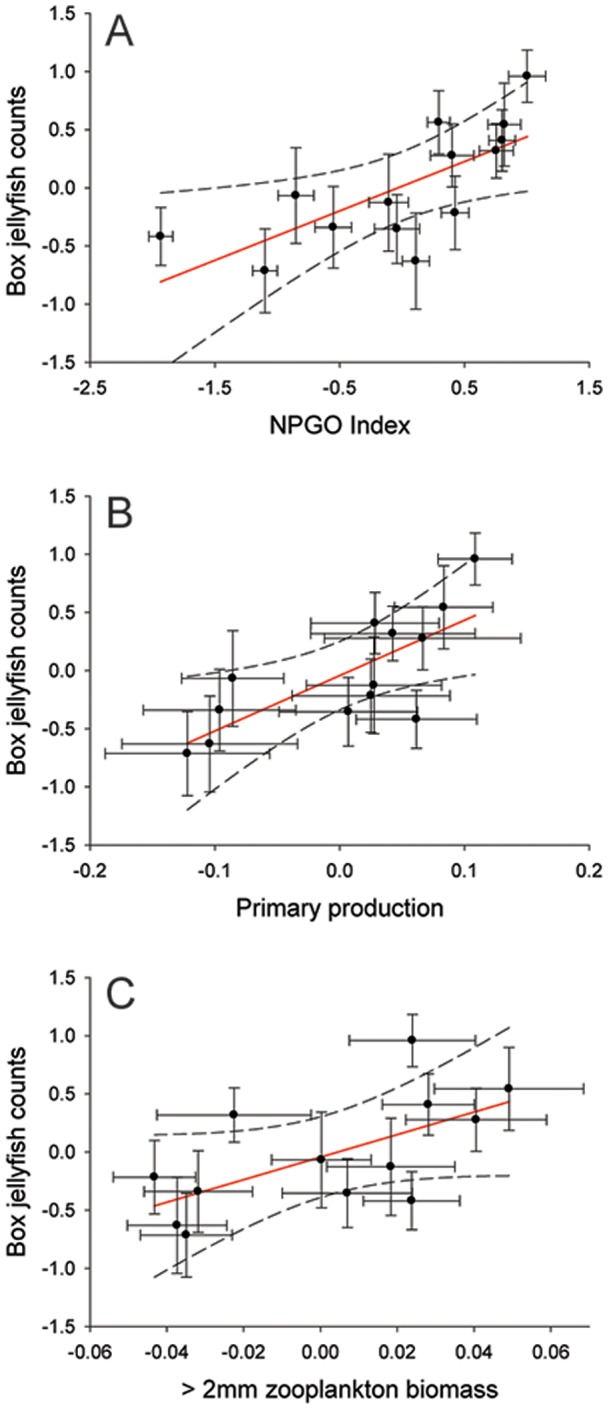
Significant relationships among box jellyfish abundance at Waikiki Beach and environmental variables. A: North Pacific Gyre Oscillation (NPGO), B: Primary production, C: zooplankton biomass within the sub-tropical North Pacific. Significant linear relationships were detected using General Regression Models (*GRM*). Biogeochemical variables were obtained at Station ALOHA, from the Hawaiian Oceanographic Time-series program (HOT) at http://hahana.soest.hawaii.edu/hot/. Solid red line represents the model fit, and dashed lines represent 99% confidence levels. Bars represent standard errors.

**Figure 6:**
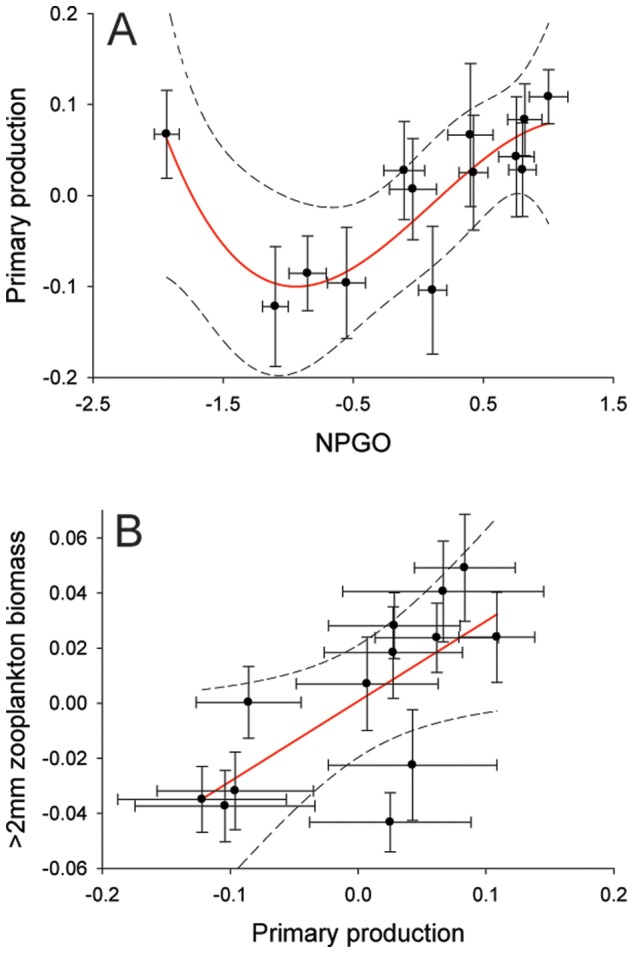
Significant relationships among environmental variables that correlated significantly with box jellyfish abundance at Waikiki Beach. A) Non-linear relationship between primary production and NPGO detected by using Generalized Additive Models (*GAM*). B) Linear relationship between zooplankton biomass and primary production detected by using General Regression Models (*GRM*). Biogeochemical variables were obtained at Station ALOHA, from the Hawaiian Oceanographic Time-series program (HOT) at http://hahana.soest.hawaii.edu/hot/. Solid red line represents the model fit, and dashed lines represent 99% confidence levels. Bars represent standard errors.

Of a total of three climate indices, 13 biogeochemical variables, and seven weather parameters analyzed with a univariate approach using the *GAM* and *GRM*, one climatic and two biogeochemical variables showed a significant relationship with beach counts (Figure 4). No significant relationships were detected between beach counts and any weather parameters examined at both monthly and annual scales (Table S3 in File S1). In addition, the *Circular-Linear* correlation analyses did not detect a significant relationship between jellyfish counts and wind direction at any time scale (Table S3 in File S1). On a monthly scale, the *GRM* indicated a weak positive, significant linear relationship between box jellyfish abundance and both the NPGO (*GRM*, *r^2^* = 0.27, *p*<0.001; Figure 4A) and >2 mm night zooplankton biomass (*GRM*, *r^2^* = 0.19, *p* = 0.036; Figure 4E). However, on an annual scale, the *GRM* revealed strong, positive linear relationships of beach counts with the NPGO (*GRM*: *F_1, 12_ = *13, *r* = 0.72, *r^2^* = 0.52; *p* = 0.003; Figures 4B & 5A), primary production (*GRM*: *F_1, 11_* = 13.5, *r* = 0.74, *r^2^* = 0.54; *p* = 0.004; Figures 4D & 5B), and >2 mm night zooplankton biomass (*GRM*: *F_1,11_* = *r* = 0.6; *r^2^* = 0.36; *p* = 0.03; Figures 4F & 5C). Significant inter-relationships (on an annual scale) were detected among variables that correlated significantly with jellyfish abundance at Waikiki Beach. Primary production (as covariate) showed a strong positive linear relationship with >2 mm night zooplankton biomass (*GRM*, *r* = 0.62; *r^2^* = 0.39; *p* = 0.01; Figure 6A). Interestingly, the *GAM* detected a strong non-linear relationship (i.e., “u” shape) between the NPGO (covariate) and primary production (*GAM, r^2^* = 0.75, non-linear *p* = 0.001; Figure 6B). Further analysis indicated that the non-linear relationship between primary production and NPGO was caused by an unusual pairwise observation for 2005. Disregarding this outlier, there was a strong, positive linear relationship between NPGO and primary production (*GRM*: *r* = 0.86; *r^2^* = 0.7; *p* = 0.0004).

## Discussion

This study represents the first long-term assessment of trends in abundance of cubozoan medusae, and the first to evaluate potential links between box jellyfish abundance and environmental conditions in a highly oligotrophic environment. Although *Alatina moseri* medusae arrived consistently at Waikiki Beach 8–12 days after full moon throughout the 14-year period, the number of medusae at the beach showed no seasonality and size (bell height) was relatively uniform throughout 2001 (Table S1 & Figure S1 in File S1). Given the lack of seasonality in the NPSG, where sea surface temperature (SST) varies only 3°C throughout the year (HOT data), this may not be surprising. In temperate regions, occurrence of medusae is usually seasonal as onset of strobilation is associated with seasonal variation in SST; however, in tropical and sub-tropical areas, jellyfish polyps typically exhibit continuous or semi-continuous strobilation thus perennial presence of medusae [54]. Continuous or semi-continuous production of medusae from polyps likely explains the lack of seasonality observed in the size of beach aggregations of box jellyfish at Waikiki. In Australia's Great Barrier Reef region, medusae of *Alatina moseri* ( =  *A. mordens* [41]) also occur year-round, and laboratory observations suggest that polyps are capable of continuous asexual reproduction, and metamorphosis into young medusae (ephyrae) is independent of water temperature variation [55]. In Puerto Rico (Caribbean Sea), polyps of *A. moseri* ( =  *Carybdea alata*) are capable of year-round asexual reproduction, and year-round occurrence of medusae of this species has been documented in this area [37]. In addition, polyps of scyphozoan jellyfish, such as *Aurelia* spp. [56] and *Mastigias* spp. [57] from tropical and sub-tropical regions, release ephyrae throughout the year in the absence of significant SST variation. Our data support earlier observations that medusae of *A. moseri* arrive to Waikiki Beach 8–12 days after each full moon [38]. Field observations indicate that box jellyfish arrive at Waikiki Beach with fully mature and active gonads, and medusae become senescent a few hours post-reproduction (sperm are broadcast, strands of fertilized eggs are expelled; AAY, unpubl. data), suggesting medusae of this species likely reproduce once in their lifetime (∼1 year, [37]). Interestingly, polyps of *A. moseri* go through a metamorphosis (at ∼75 days of age, raised at 26–29°C in the laboratory) in which the whole polyp becomes a single young medusa, and no remnant is left behind [37]. Therefore, continuous or semi-continuous metamorphosis from polyps to medusae may be required to maintain the adult medusae cohort over time, while monthly sexual reproductive events may be necessary to sustain an ephemeral polyp population.

Box jellyfish abundance at Waikiki Beach showed neither net increase nor net decrease throughout the 14-year period of study; instead, it showed dramatic monthly and annual variability in a cyclical, non-linear pattern with intermittent periods (pulses) of increase (positive phase) and decrease (negative phase) (Figures 2 & 3). These results are consistent with recent long-term studies suggesting that jellyfish populations, on a global scale, oscillate over different time scales with no apparent long-term trend [20], [30]. The arrival of box jellyfish medusae at Waikiki Beach was recurrent with consistent, predictable timing, however, monthly aggregation size varied substantially. Thus the model (*GAM*) showed a poor fit (12%) with monthly beach counts from Jan 1998 to Dec 2011. Month to month comparison of jellyfish beach counts are notorious for their high temporal variability [58], [59], and a number of physical factors, including waves, currents, topography, individual behavior and life history, could all play a role in the monthly variation in numbers of medusae at the beach [58], [59], [60]. Predictability of our model, however, increased (53%) when applied to annual mean beach counts, which are typically considered a more reliable proxy for population abundance for beach counts [61], [62].

Our understanding of inter-annual changes in abundance of box jellyfish at Waikiki Beach was enhanced by comparison with long-term local and regional environmental heterogeneity: counts at Waikiki Beach did not correlate significantly with any of the local weather parameters studied (Table S3 in File S1), whereas years of high jellyfish beach counts coincided with years of elevated NPGO (North Pacific Gyre Oscillation) index, primary production, and macro-zooplankton abundance. Thus, fluctuations in abundance of *Alatina moseri* medusae at Waikiki Beach appear to follow decadal climatic oscillations occurring within the sub-tropical North Pacific, independent of local weather conditions (wind speed, wind direction, air temperature, rainfall, cloud cover, humidity, and atmospheric pressure; Table S3 in File S1). Beach counts showed a strong, positive correlation with the North Pacific Gyre Oscillation (NPGO), while no significant relationship was detected with any other large-scale climate force, such as the Pacific decadal Oscillation (PDO) and the Multivariate ENSO Index (MEI). Unlike the PDO and MEI [42], [43], the NPGO is associated with variation in wind-driven circulation including upwelling and horizontal advection at both regional and basin scales, and physical processes controlling salinity and nutrient concentrations [44], which in turn drive changes in phytoplankton levels, leading to variability at higher trophic levels. Thus the NPGO is an indicator of fluctuations in the mechanisms controlling planktonic ecosystem dynamics [44]. The NPGO is considered the primary climate index linked to decadal biological variability in the NPSG. Recent studies [63], [64] using biogeochemical ocean circulation models, showed NPGO to be the only climate index significantly correlated with primary production at Station ALOHA, in agreement with results of this study. In fact, neither PDO nor MEI was significantly correlated with beach counts or with any biogeochemical variable that showed a significant relationship with box jellyfish abundance. Although the actual mechanism has not been determined, Dave and Lozier (2010) [64] suggest that the correlation between the NPGO and primary productivity in the NPSG may be driven by changes in diazotrophic activity (N_2_-fixation). Approximately 80% of all nitrogen fixation at Station ALOHA occurs within 60 m of the surface, and evidence suggests that diazotrophs account for up to half of vertically integrated new production in the region [65], [66], [67]. An increase in N-fixation activity translates into introduction of new nitrogen in the system, which can in turn cause an increase in zooplankton abundance via direct trophic transfer [68], [69].

The strong positive relationship between annual primary production and zooplankton biomass (secondary production) observed in this study may reflect trophic interactions previously observed at Station ALOHA [69], where copepods comprise the dominant meso-zooplankton taxa (∼175 species) as well as the primary consumers. Such periods of high primary production can support elevated secondary productivity [69]. In this study, we detected a strong positive correlation between box jellyfish counts and zooplankton, suggesting that abundance of the box jellyfish *Alatina moseri* at Waikiki Beach could be influenced by regional prey availability. Cubomedusae are predators that feed on a variety meso- and macro-zooplankton taxa [70], [71], [72], [73]. Although detailed prey preference data for *A. moseri* is not currently available, preliminary molecular analysis of gut contents has confirmed the presence of oceanic meso- and macro-zooplankton taxa (Holland et al., in prep). Jellyfish are known to increase growth rates and reach larger adult sizes in response to increased food availability [74], and since body size positively correlates with fecundity in jellyfish, more eggs (and more larvae) are produced when food is readily available [75], [76], potentially increasing larval recruitment [54]. At the same time, increased food availability in the water column can also affect the amount of food reaching the benthos [77], [78], [79], and jellyfish polyps are known to increase budding rates (i.e., asexual production of new polyps) in response to an increase in food supply [80], [81], [82], [83], potentially translating into higher numbers of medusae released via strobilation during periods with high zooplankton biomass compared to years of low zooplankton biomass. In polyps of the box jellyfish *A. moseri* ( =  *A. mordens*, [41]) increased feeding frequency resulted in increased budding rates [55], with similar results obtained for a related species [84]. As previously mentioned, abundance of *A. moseri* medusae is not seasonal in Hawaii (US, Waikiki Beach), the Caribbean (Puerto Rico) [37], and the Great Barrier Reef (Australia) [55], probably due to continuous production of medusae via polyp metamorphosis, which may account for the lack of a significant lag between jellyfish abundance of jellies arriving at the beach and zooplankton biomass observed in this study.

In spite of the distance between collection localities for jellyfish (Waikiki) and biogeochemical variables (Station ALOHA: 22.45°N, 158°W, ∼200 km north of Oahu), correlations are of value for a number of reasons. Station ALOHA was selected because it is representative of oceanographic processes occurring throughout the NPSG [45]. In fact, *Alatina moseri* is one of the few pelagic cubozoan medusae [41], and is seen near shore for only 2–4 days, 8–12 days after each full moon. During non-aggregation periods, medusae have been collected from near the surface offshore, in waters several hundred meters deep [85], and on the day of arrival at the beach, *A. moseri* medusae have been observed ∼2 km south of Waikiki Beach actively swimming straight towards the shore against or across currents (AAY, unpubl. data), evidencing the swimming capabilities of large cubozoans [86]. In addition, the Hawaiian Islands were formed via volcanic hotspot located near the center of the NPSG, where oceanic conditions occur close to shore, and depths >400 m are reached within 5 km of the coastline. Thus, regional offshore climatic and biogeochemical processes recorded at Station ALOHA likely have relevance to inter-annual fluctuations in numbers of cubozoan medusae arriving at Waikiki Beach. Given the fact that only mature *A. moseri* medusae arrive at Waikiki Beach from offshore 8–12 days after each full moon, annual fluctuation in beach counts could reflect inter-annual changes in adult off-shore population size. However, the possibility that beach counts do not reflect offshore abundance cannot be ruled out. In the Celtic Sea, for example, medusae of *Chrysaora hysoscella* were shown to be abundant offshore but were rarely observed at the shoreline [58]. However, our observations of *A. moseri* actively swimming towards the beach during the ebbing tide, against as well as across surface currents and tidal flow, contrasts with passive stranding events observed for instance, in scyphozoan medusae [58], [59]. Lunar cycles have previously been shown to affect swimming behavior of jellyfish [33] and reproductive cycles of marine Cnidaria [87]. We suggest that the moon cycle may play a role in synchrony of gonad development and timing of *Alatina moseri* arrival at the shore, while environmental variables, such as regional food availability may play a role in fluctuations in Waikiki beach counts.

Long-term, optimally multi-decadal studies are necessary to understand complex ecological factors driving population fluxes in pelagic marine taxa. Box jellyfish aggregations are a major, and poorly understood public safety hazard in populated coastal areas, and present persistent challenges to resource managers and lifeguard services. Only through an enhanced understanding of the relevant ecological drivers will we be able to generate predictive models to minimize public health impacts. Perhaps the most important finding here is that annual Waikiki beach counts appear to be influenced by fluctuation in environmental conditions within the NPSG, including climate (NPGO) and certain biological variables, independent of local weather conditions. These findings suggest that food availability within the NPSG may be an important predictor of inter-annual fluctuations in abundance of *A. moseri* medusae during beach aggregations in Hawaii, likely via bottom-up control. Additional studies will be required to test whether observed fluctuations in beach counts correspond to abundance offshore for Waikiki box jellyfish. Our work will continue to focus on the oscillating aggregation patterns revealed in this study, and on elucidation of the environmental mechanisms governing fluctuations of this important and poorly understood species.

## Supporting Information

File S1 Monthly average bell height of Alatina moseri medusae collected at Waikiki Beach throughout 2001 (Table S1), bell height frequency distribution of Alatina moseri medusae collected at Waikiki Beach throughout 2001 (Figure S1), monthly counts (raw data) of Alatina moseri medusae recorded at Waikiki Beach from January 1998 to December 2011 (Table S2), and non-significant correlations between numbers of Alatina moseri medusae at Waikiki Beach and climate indices, biogeochemical variables, and weather parameters (Table S3).
(DOCX)
