# Supplementary material for: Long-Term Fluctuations in Circalunar Beach Aggregations of the Box Jellyfish Alatina moseri in Hawaii, with Links to Environmental Variability
Source: PLoS One. 2013 Oct 23;8(10):e77039. doi: 10.1371/journal.pone.0077039 (PMC3806728; doi:10.1371/journal.pone.0077039)
Supplement: File S1 — Monthly average bell height of Alatina moseri medusae collected at Waikiki Beach throughout 2001 (Table S1), bell height frequency distribution of Alatina moseri medusae collected at Waikiki Beach throughout 2001 (Figure S1), monthly counts (raw data) of Alatina moseri medusae recorded at Waikiki Beach from January 1998 to December 2011 (Table S2), and non-significant correlations between numbers of Alatina moseri medusae at Waikiki Beach and climate indices, biogeochemical variables, and weather parameters (Table S3). (DOCX) [file pone.0077039.s001.docx]

| *Month* | *Mean Bell Height (mm)* | *SE* | *-0.95* | *+0.95* | *n* |
| --- | --- | --- | --- | --- | --- |
| *Jan* | 61.02 | 1.16 | 58.74 | 63.30 | 52 |
| *Feb* | 60.02 | 0.77 | 58.50 | 61.53 | 118 |
| *Mar* | 63.90 | 0.45 | 63.03 | 64.78 | 353 |
| *Apr* | 62.65 | 0.42 | 61.82 | 63.48 | 391 |
| *May* | 66.78 | 0.53 | 65.74 | 67.82 | 251 |
| *Jun* | 62.09 | 0.27 | 61.57 | 62.61 | 1000 |
| *Jul* | 71.34 | 0.38 | 70.60 | 72.08 | 493 |
| *Aug* | 67.32 | 0.42 | 66.49 | 68.14 | 400 |
| *Sep* | 62.28 | 0.44 | 61.41 | 63.14 | 361 |
| *Oct* | 65.20 | 0.50 | 64.23 | 66.17 | 286 |
| *Nov* | 58.82 | 0.42 | 58.00 | 59.65 | 400 |
| *Dec* | 68.67 | 0.76 | 67.18 | 70.15 | 123 |

Table S1. Mean bell height of *Alatina moseri* medusae collected in 2001.

Figure S1. Histogram representing the distribution of bell height in medusae of *Alatina moseri* collected at Waikiki Beach in 2001 (*n* = 4228).

Table S2. Monthly number of *Alatina moseri* medusae collected at Waikiki Beach from Jan 1998 to Dec 2011. Some years had 13 waning crescent lunar phases (8-12 days after full moon) instead of 12; thereby, two jellyfish arrivals happened within the same month in five instances (indicated in yellow). In those cases, we used the average jellyfish counts.

|  | *Monthly* | *Annual* | *1-Year Lag* |
| --- | --- | --- | --- |
| ***Climatic variables*** |  |  |  |
| *Multivariate ENSO Index (MEI)* | -0.16 (p= 0.10) | -0.49 (p= 0.09) | -0.47 (p= 0.10) |
| *Pacific Decadal Oscillation (PDO)* | -0.10 (p= 0.21) | -0.52 (p= 0.08) | -0.36 (p= 0.24) |
| *North Pacific Gyre Oscillation (NPGO)* | (*significant*) | (*significant*) | 0.56 (p= 0.06) |
|  |  |  |  |
| ***Biogeochemical variables*** |  |  |  |
| *Temp (^o^C)* | -0.004 (p= 0.9) | 0.09 (p= 0.77) | -0.28 (p= 0.36) |
| *Salinity (ppt)* | 0.04 (p= 0.70) | 0.14 (p= 0.64) | 0.47 (p= 0.11) |
| *Particulate Nitrogen (mmol.m^-2^)* | -0.14 (p= 0.12) | -0.29 (p= 0.34) | -0.51 (p= 0.07) |
| *Particulate Carbon (mmol.m^-2^)* | -0.07 (p= 0.44) | -0.19 (p= 0.43) | -0.12 (p= 0.69) |
| *Chlorophyll a (mg m^-2^)* | 0.02 (p= 0.84) | -0.29 (p= 0.34) | 0.07 (p= 0.83) |
| *Prochlorococcus (# x10 ^11^)* | -0.13 (p= 0.16) | -0.24 (p= 0.44) | 0.28 (p= 0.34) |
| *Eukaryotes (# x 10 ^11^)* | 0.02 (p= 0.83) | 0.26 (p= 0.46) | -0.39 (p= 0.19) |
| *Heterotrophic bacteria (# x 10 ^11^)* | -0.17 (p= 0.09) | -0.23 (p= 0.39) | -0.37 (p= 0.21) |
| *< 2mm (night) zoop. biomass (gDW m^-2^)* | 0.14 (p= 0.11) | 0.21 (p= 0.47) | -0.29 (p= 0.32) |
| *< 2mm (day) zoopl. biomass (gDW m^-2^)* | 0.09 (p= 0.32) | -0.16 (p= 0.61) | -0.33 (p= 0.27) |
| *> 2mm (day) zoopl. biomass (gDW m^-2^)* | 0.07 (p= 0.58) | 0.32 (p= 0.21) | -0.31 (p= 0.29) |
| *> 2mm (night) zoopl. biomass (gDW m^-2^)* | (*significant*) | (*significant*) | -0.21 (p= 0.49) |
| *Primary production (mgC m^-2^ day^-1^)* | 0.05 (p = 0.56) | (*significant*) | 0.1 (p= 0.75) |
|  |  |  |  |
| ***Local weather parameters*** |  |  |  |
| *Air temperature (^o^C)* | -0.04 (p= 0.62) | 0.27 (p= 0.34) | -0.28 (p= 0.34) |
| *Atmospheric pressure (mmHg)* | 0.03 (p= 0.71) | 0.13 (p= 0.67) | 0.22 (p= 0.47) |
| *Rainfall (mm)* | 0.05 (p= 0.53) | -0.22 (p= 0.45) | -0.43 (p= 0.14) |
| *Cloud cover (%)* | -0.01 (p= 0.81) | -0.37 (p= 0.23) | -0.49 (p= 0.08) |
| *Wind speed (km/h)* | -0.06 (p= 0.46) | -0.08 (p= 0.78) | 0.37 (p= 0.22) |
| *Wind direction (Degrees)* | 0.11 (p= 0.16) | 0.43 (p= 0.15) | -0.40 (p= 0.17) |
| *Humidity (%)* | 0.09 (p= 0.24) | 0.24 (p= 0.40) | -0.01 (p= 0.97) |

Table S3. Table showing the correlation coefficient of all non-significant relationships detected between environmental variables and numbers of box jellyfish counted at Waikiki Beach (Jan 1998 to Dec 2011). Coefficients and *p*-values were obtained using linear regressions (via General Regression Models after a non-significant relationship detected by the GAM; see methods), except for wind direction for which coefficients were obtained by a Circular-Linear correlation analysis. All variables were log-transformed and detrended before the analysis, except for wind direction (see Methods).
